# Case Report: Compromised Airway Following Anesthesia and Its Correlation With the Use of ACE Inhibitors—An Unexpected Clinical Event and Review of Literature

**DOI:** 10.3389/fsurg.2021.631456

**Published:** 2021-03-22

**Authors:** Sanjeev Mohanty, Ashish Bangaari, Kumaran Gnanasekaran

**Affiliations:** ^1^MGM Health Care, Chennai, India; ^2^Department of Anaesthesia, MIOT International, Chennai, India; ^3^MIOT International, Chennai, India

**Keywords:** angioedema, bradykinin, angiotensin-converting enzyme, tongue oedema, airway

## Abstract

Angiotensin-converting enzyme inhibitors associated angioedema involving the upper aerodigestive tract is indisputably a hazardous airway condition which is clinically poorly recognized and frequently underestimated. We describe and present case of a 70-year old man on ramipril who developed massive tongue swelling post-operatively after unremarkable laryngeal mask anaesthesia which was fortuitously managed conservatively. High index of suspicion, timely recognition and knowledge of pathophysiology and the clinical course should guide airway and further supportive management in these patients.

## Introduction

A known risk for patients taking angiotensin-converting enzyme inhibitors (ACE-I) is angioedema caused by bradykinin, which can involve the face, lips, tongue, and upper airway. Since the clinical presentation is variable and unpredictable, with no possibilities to prevent and predict its development, the diagnosis can be delayed, potentially translating into increased patient suffering and risk of suffocation. The clinical uncertainty and the rapidity of airway compromise raised an alarm and thus was reported to raise the awareness of health care professionals sharing the common unified airway and propose preventive attitudes for discussion that could improve the perioperative safety of patients.

## Case Profile

**Figure 1:**
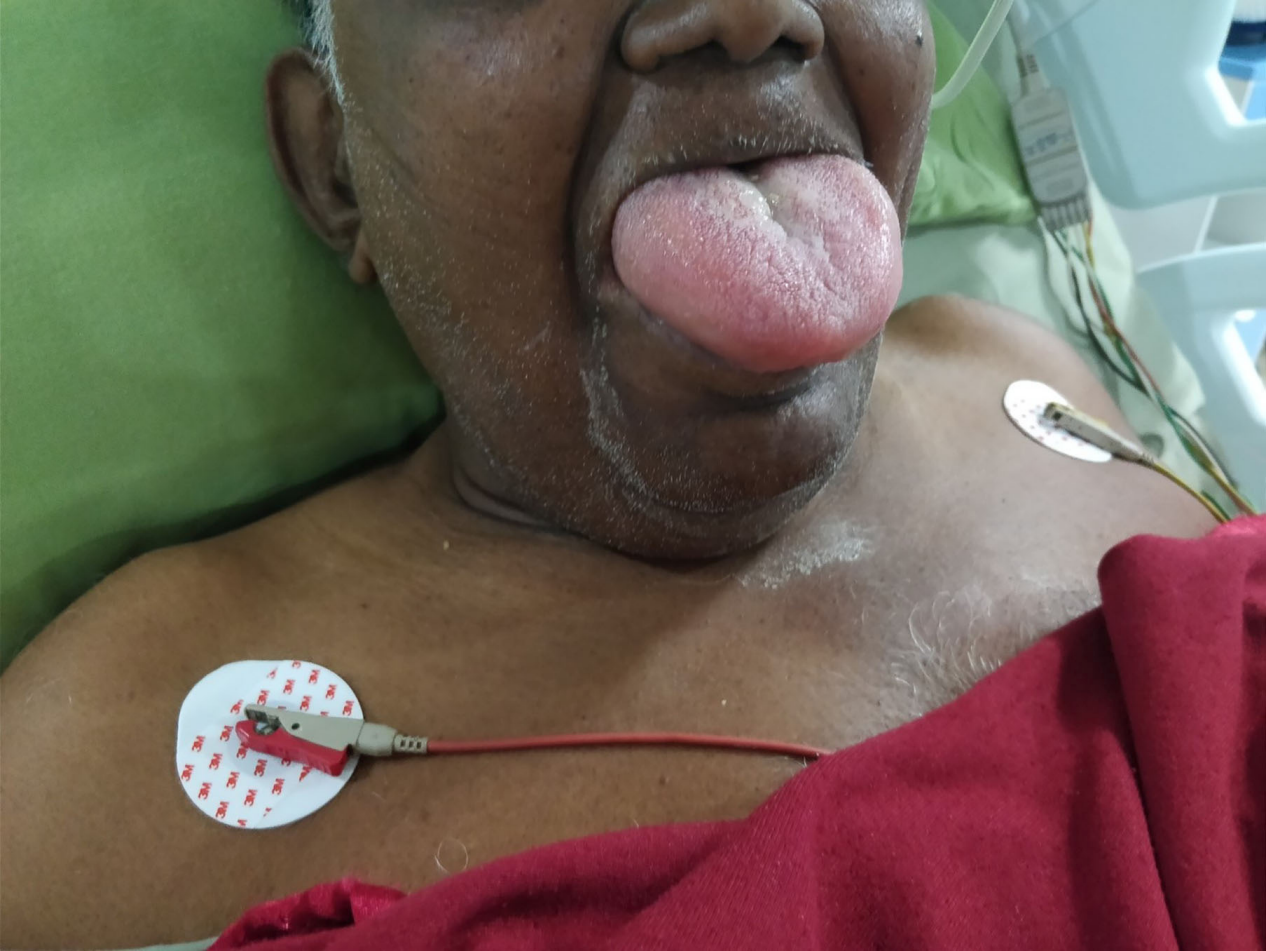
Post-operative massive tongue oedema.

Our patient, 70 years/male known hypertensive for 6 years on Ramipril 5 mg and Telmisartan 40 mg with no known drug allergy, underwent a minor urology procedure (20 min) under propofol sedation with four size classical laryngeal mask airway (LMA) under spontaneous respiration. Anesthesia and recovery were uneventful with stable hemodynamics. Non-steroidal anti-inflammatory agents were not administered perioperatively. After 4 h, the patient's tongue was noted to be slightly larger than normal without any complaints of urticaria or pruritis. Within the next 2 h, tongue swelling rapidly progressed to occupy the entire oral cavity and forced his mouth to open widely (Figure 1). Saliva was dripping from his mouth as he was unable to clear his secretion, with impaired speech, without any respiratory distress or dysphonia. On probing further, his family revealed history of two episodes of similar sized swollen tongue managed by intravenous steroids and nebulization by the family physician 2 years and 6 months back without any complications. There was no family history of similar symptoms. A clinical assessment of possible angiotensin-converting enzyme inhibitors-induced angioedema (ACEI-IA) was made.

**Figure 2:**
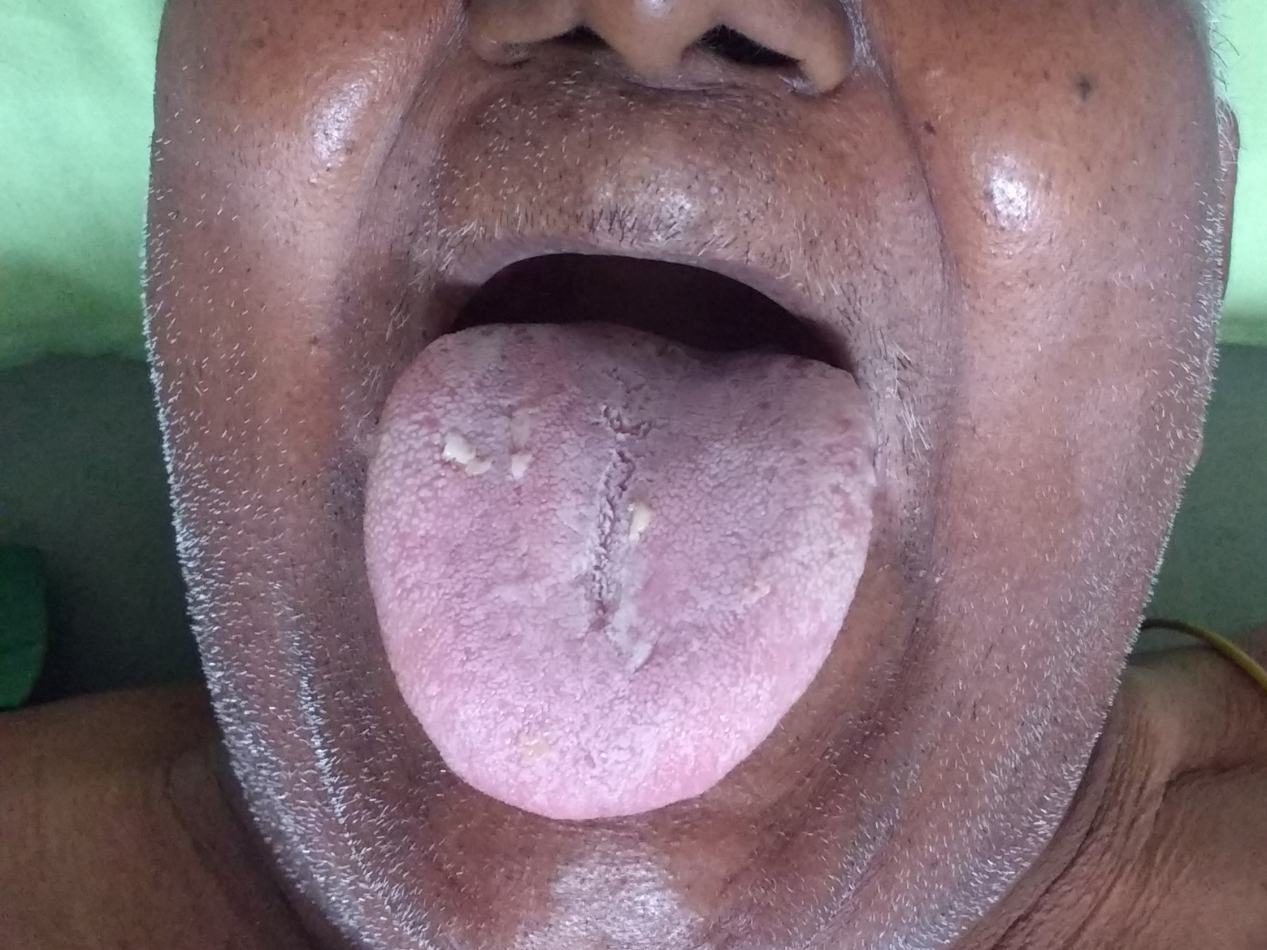
Overnight resolution of tongue oedema.

He was transferred to the intensive care unit, where he was propped up 60 degrees and administered IV steroids, anti-histaminic, adrenaline nebulization, and oxygen. Overnight, he was kept fasting, with a difficult airway cart and a tracheostomy kit bedside. By the next day, his tongue became normal in size, and he could speak and had oral intake of liquids (Figure 2). His daily medicines were reviewed, and ramipril and telmisartan were suspended. Subsequent postoperative course was uneventful.

## Discussion

Acute postoperative tongue edema is usually a consequence of mechanical trauma, and vascular and lymphatic obstruction due to hard bite block or tight throat packs, substance allergy (local), hypersensitivity to intravenous agent, abscess, infection, acute urticaria, or massive postoperative fluid loading (1). Angioedema characterized by a rapid, localized, temporary, non-inflammatory area of swelling, affecting the tongue, face, and subcutaneous tissue of the upper respiratory tract can be triggered by trauma, airway instrumentation, and infection in susceptible subjects (2).

Pathophysiology of angioedema involves a vascular reaction of deep dermal/subcutaneous tissues or mucosal/submucosal tissues with localized increased permeability of blood vessels resulting in tissue swelling (3). It is classified as allergic or histamine mediated, and non-allergic/drug induced which is bradykinin mediated where urticaria and itching is absent. A wide scope of drugs can be responsible for inducing angioedema; ACE-I (most common), non-steroidal anti-inflammatory drugs, antibiotics, proton pump inhibitor, radiocontrast media, fibrinolytic agents, angiotensin II receptor antagonist, beta-adrenolytics, and psychotropic drugs (4).

ACEI-IA is a rare side effect (0.2–0.5%), but the large number of people taking these medications makes it the leading cause of drug-induced angioedema (5). The incidence is underestimated as many physicians misdiagnosed it as an anaphylactic reaction, which delays the diagnosis, leading to poor initial management, deleterious to the patient (6). Possible risk factors include female gender, black race, previous history of angioedema, smoking, seasonal allergies, recent initiation of ACE-I, and immunosuppression (4). It is likely that complex genetic and other humoral factors such as nitric oxide, interleukin-1, tumor necrosis factor along with several mediators like bradykinin, Des-Arg9-BK (metabolite of bradykinin), histamine, substance P, and prostaglandins are involved in the pathogenesis (7, 8). Defects in the secondary bradykinin metabolic enzymes aminopeptidase P, kininase I, neutral endopeptidase, and dipeptidyl peptidase-4 have also been suggested as a mechanism (9). However, at present, there is no standardized genetic or laboratory evaluation that can identify in advance individual patients who are at risk for developing ACE-IA (10).

The clinical presentation is variable, course is unpredictable, and attacks can recur erratically with variation in severity. In most cases, angioedema occurs during the first week of exposure, although it may occur any time during the course of therapy from hours to years after treatment. The symptoms in most cases are mild and regress spontaneously without complications even when the patient continues to take ACE-I. Swelling usually develops over minutes to hours, peaks, and then resolves over 24 to 72 h, although complete resolution may take days in some cases (11). The pathophysiological reason for this pattern is not known. ACE-IA is a class effect and is not agent- or dose-dependent, and any angioedema occurring during a treatment with ACE-I should be considered as a bradykinin-mediated angioedema (12).

The standard of care for ACE-IA has been non-specific and consists of discontinuation of the ACE inhibitor, observation, and airway management. Up to 20% of cases may present with dysphonia and stridor with rapid progression to life-threatening airway obstruction (10). Therefore, the airway should always be evaluated by an ear–nose–throat (ENT) specialist in these cases by fiberoptic nasal endoscopy to formulate airway management plan (13). Involvement of soft tissue neck may make cricothyrotomy, trans-tracheal jet ventilation, and tracheostomy difficult (10).

The non-allergic nature of the reaction renders traditional therapies (corticosteroids and antihistamines) ineffective yet are often administered empirically (10, 14). Several therapies effective in aborting attacks of angioedema in hereditary angioedema (HAE), the best-studied form of bradykinin-mediated angioedema, have been proposed for ACE-IA. Medications like Ecallantide (inhibit conversion of kininogen into kinins) or Icatibant (selective bradykinin B2 receptor antagonist), C1 inhibitor concentrate, and fresh frozen plasma have been suggested with mixed results and outcome (15). Tranexamic acid decreases production of bradykinin by inhibiting plasminogen and has shown promising results in preventing HAE attacks. It may be a beneficial treatment modality in the management of ACE-IA and warrants further investigation (16). The current therapeutic options of ACE-IA are insufficient and unsatisfying as only off-label therapy options are available and previous study results are incongruent (5). Despite the potential severity of this disease state, there is no consensus regarding emergency pharmacological management of these episodes (13).

Since our laryngeal mask usage was atraumatic, without tongue manipulation or mayo cannula usage, and for a minimal duration, it was an *unlikely* cause of worsening tongue edema. Previous similar outpatient episodes also corroborated with the possibility of ACEI-IA. HAE commonly occurs at adolescence or childhood with positive family history, which were missing in our case even though there were recurrent angioedema episodes at old age. Retrospectively thinking, we probably could have investigated for serum C1-esterase levels and possible genetic deficiency to rule out HAE. Elderly, obesity, and airway instrumentation may have contributed as predisposing factors in our patient. Therefore, patients with recent ACE-I intake probably should have postoperative surveillance, discharge counseling, and accessibility to emergency care units after receiving general anesthesia. Unfortunately, there is still missing consensus about perioperative surveillance guidelines in patients taking ACE-I (17). Physician error with failure to document in the medical records, the suspicion of ACEI-IA, and failure to consider risk in prescribing ACE-I after an episode of angioedema had occurred are a common cause of recurrent ACEI-IA (18). Consensus guidelines emphasize close monitoring, airway intervention as required, and lifetime avoidance of all ACE-I (14).

## Conclusion

Perioperative ACE-IA is a rare, underreported, and intimidating event that is potentially life threatening. ACE-IA should be a differential diagnosis in any postoperative patient presenting with acute upper aerodigestive tract swelling without pruritis and with present or previous ACE-I treatment. An experienced anesthetist, intensivist, and ENT surgeon should be part of the team managing ACE-IA. Timely recognition, clinical judgment, and knowledge of its self-limiting course should guide airway and supportive management. Postoperative surveillance, counseling, and accessibility to emergency care units should be covered in discharge policy of patients on ACE-I.

## Data Availability Statement

The original contributions presented in the study are included in the article/supplementary material, further inquiries can be directed to the corresponding author/s.

## Ethics Statement

Written informed consent was obtained from the individual(s) for the publication of any potentially identifiable images or data included in this article.

## Author Contributions

All authors listed have made a substantial, direct and intellectual contribution to the work, and approved it for publication.

## Conflict of Interest

The authors declare that the research was conducted in the absence of any commercial or financial relationships that could be construed as a potential conflict of interest.
